# Distinct ET_A_ Receptor Binding Mode of Macitentan As Determined by Site Directed Mutagenesis

**DOI:** 10.1371/journal.pone.0107809

**Published:** 2014-09-16

**Authors:** John Gatfield, Celia Mueller Grandjean, Daniel Bur, Martin H. Bolli, Oliver Nayler

**Affiliations:** Actelion Pharmaceuticals Ltd., Allschwil, Switzerland; Vanderbilt University Medical Center, United States of America

## Abstract

The competitive endothelin receptor antagonists (ERA) bosentan and ambrisentan, which have long been approved for the treatment of pulmonary arterial hypertension, are characterized by very short (1 min) occupancy half-lives at the ET_A_ receptor. The novel ERA macitentan, displays a 20-fold increased receptor occupancy half-life, causing insurmountable antagonism of ET-1-induced signaling in pulmonary arterial smooth muscle cells. We show here that the slow ET_A_ receptor dissociation rate of macitentan was shared with a set of structural analogs, whereas compounds structurally related to bosentan displayed fast dissociation kinetics. NMR analysis showed that macitentan adopts a compact structure in aqueous solution and molecular modeling suggests that this conformation tightly fits into a well-defined ET_A_ receptor binding pocket. In contrast the structurally different and negatively charged bosentan-type molecules only partially filled this pocket and expanded into an extended endothelin binding site. To further investigate these different ET_A_ receptor-antagonist interaction modes, we performed functional studies using ET_A_ receptor variants harboring amino acid point mutations in the presumed ERA interaction site. Three ET_A_ receptor residues significantly and differentially affected ERA activity: Mutation R326Q did not affect the antagonist activity of macitentan, however the potencies of bosentan and ambrisentan were significantly reduced; mutation L322A rendered macitentan less potent, whereas bosentan and ambrisentan were unaffected; mutation I355A significantly reduced bosentan potency, but not ambrisentan and macitentan potencies. This suggests that – in contrast to bosentan and ambrisentan - macitentan-ET_A_ receptor binding is not dependent on strong charge-charge interactions, but depends predominantly on hydrophobic interactions. This different binding mode could be the reason for macitentan's sustained target occupancy and insurmountable antagonism.

## Introduction

Endothelins (endothelin-1, endothelin-2 and endothelin-3) are vasoactive peptides mainly produced by endothelial cells, but also by smooth muscle cells, fibroblasts and macrophages. Endothelin-1 (ET-1), which is known to be a potent and long lasting vasoconstrictor, also acts as a mitogen, angiogenic factor, mediator of fibrosis and inflammation, and has a pathogenic role in a variety of cardiovascular disorders [1]. ET-1 responses are mediated via activation of two homologous G protein-coupled receptor subtypes, endothelin receptor subtype A (ET_A_) and endothelin receptor subtype B (ET_B_) [2], [3]. Both receptor subtypes activate Gq protein-mediated pathways leading to phospholipase Cβ and PKC activation and increased intracellular calcium concentrations [4]. In lung tissue of patients suffering from pulmonary arterial hypertension (PAH) ET-1 concentrations are elevated [5], [6]. These increases in local ET-1 concentrations cause activation of endothelin receptors in pulmonary arterial smooth muscle cells (PASMC). Consequently, increased intracellular calcium levels promote cytoskeletal contraction and cell proliferation [4], [5], [7] and thereby mediate persistent constriction and remodeling of pulmonary arteries, two hallmarks of PAH pathology [8]–[11]. The central pathogenic role of ET-1 in PAH has been demonstrated in several clinical trials evaluating different endothelin receptor antagonists (ERAs) [12].

Two approved ERAs have been used during the past years to treat patients with PAH, bosentan (Tracleer) and ambrisentan (Letairis/Volibris) [13], [14]. In 2013, the novel dual ERA macitentan (Opsumit) [15] demonstrated efficacy in a long-term event-driven phase 3 clinical trial [16] and has recently received marketing authorisation in many countries. Macitentan is significantly less acidic (pKa = 6.2) than bosentan (pKa = 5.1) and ambrisentan (pKa = 3.5) and more lipophilic (logD = 2.9 compared to logD = −0.4 for ambrisentan and logD = 1.3 for bosentan) [17]. Recent work has revealed a significant difference of endothelin receptor binding kinetics between macitentan, bosentan and ambrisentan [18]. Experiments in pulmonary arterial smooth muscle cells (PASMC) showed sustained ET_A_ receptor occupancy by macitentan (t_1/2_∼17 min) and short-lived receptor occupancy by ambrisentan and bosentan (t_1/2_∼1 min). As seen for many competitive antagonists with sustained receptor occupancy [19], this led to insurmountable ET_A_ receptor antagonism by macitentan in PASMC, i.e. macitentan blocked ET-1 signaling at high agonist concentration, whereas bosentan and ambrisentan were ineffective in these conditions [18].

The kinetic behavior of bosentan and ambrisentan is typical of high-affinity compounds with diffusion-controlled receptor interaction. Such compounds display fast receptor association and dissociation rates, and a typical receptor occupancy half-life for a diffusion-controlled 1-nM compound is less than 10 minutes (bosentan and ambrisentan have half-lives of 1 min!). In contrast, the kinetic behavior displayed by macitentan is typical of compounds for which factors beyond diffusion are limiting receptor association [20]–[22]. Such compounds are characterized by slower receptor association as well as slower dissociation rates.

Mechanisms potentially affecting ligand-receptor interaction kinetics include 1) the need for conformational changes of ligand and/or receptor during binding, 2) the type of interaction (electrostatic versus hydrophobic), and 3) the ease of release and re-entry of water molecules from/into the binding site.

In this study we characterized affinities and kinetic properties of various macitentan and bosentan analogs and show that the different kinetic properties originally found for macitentan and bosentan are replicated by structurally close analogs. We further demonstrate that macitentan prefers a compact conformation in aqueous media with minimized hydrophobic surface. Supporting molecular modeling studies suggest that this compact conformation optimally occupies a sub-pocket of the ET-1 binding site of the ET_A_ receptor. Finally, functional studies in point-mutated ET_A_ receptor variants revealed that the interaction of macitentan and the ET_A_ receptor does not depend on charge-charge interactions, but is dominated by tight hydrophobic interactions that result from an optimal shape-match between antagonist and binding pocket.

We conclude that the topological and physicochemical properties of macitentan contribute to its slower receptor interaction kinetics, which differentiate is from the other two ERAs.

## Results

### Kinetic properties of macitentan and a diverse set of structural analogs

**Figure 1:**
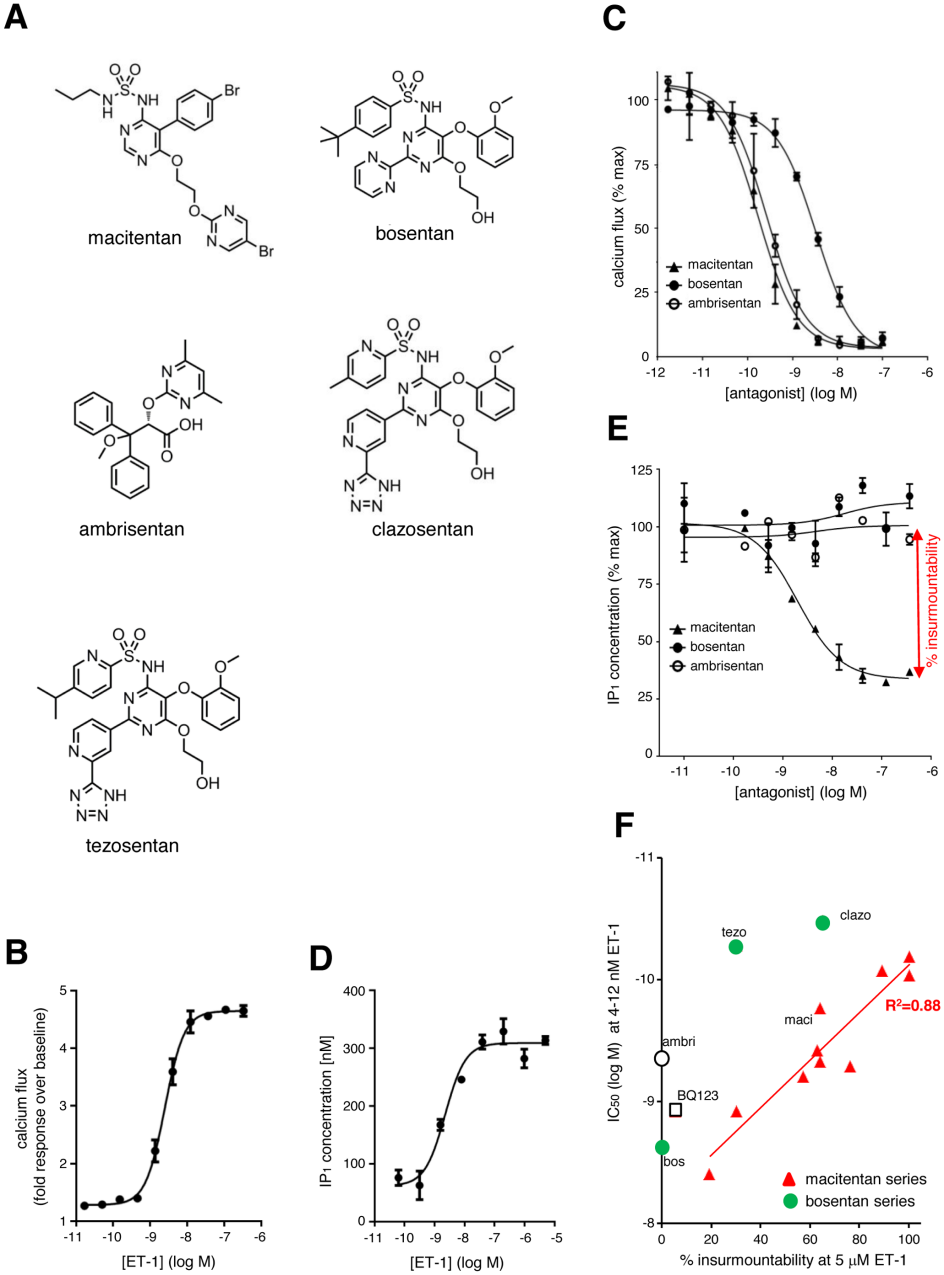
Affinity and insurmountability of macitentan analogs, bosentan analogs, ambrisentan and BQ123 analyzed using ET-1 signaling assays in PASMCs. (A) Chemical structures of macitentan, bosentan, ambrisentan, clazosentan and tezosentan. (B) Representative concentration-response curve for ET-1 in calcium flux assays. Values represent the average of duplicates +/− SD. (C) Antagonist affinity: concentration-response curves for macitentan, bosentan and ambrisentan determined in ET-1-induced calcium flux assays using ET-1 (EC_80_). Results of a representative experiment out of n = 6 independent experiments are shown. Values represent averages of duplicates +/− SD. (D) Representative concentration-response curve for ET-1 in IP_1_ accumulation assays. Values represent the average of duplicates +/− SD. (E) Antagonist insurmountability: concentration-response curves for macitentan, bosentan and ambrisentan determined in ET-1-induced IP_1_ accumulation assays using 5 µM ET-1. The degree of insurmountability of macitentan is indicated by the double-headed arrow. Results of a representative experiment out of n = 7 independent experiments are shown. Values represent averages of duplicates +/− SD. (F) Correlation of affinity (log IC_50_ (M), determined in calcium flux assays) and % insurmountability (determined in IP_1_ accumulation assays) for the tested macitentan analogs, bosentan analogs, BQ123 and ambrisentan. Linear regression for the macitentan series (red line) was performed and the R^2^ values are indicated.

**Figure 2:**
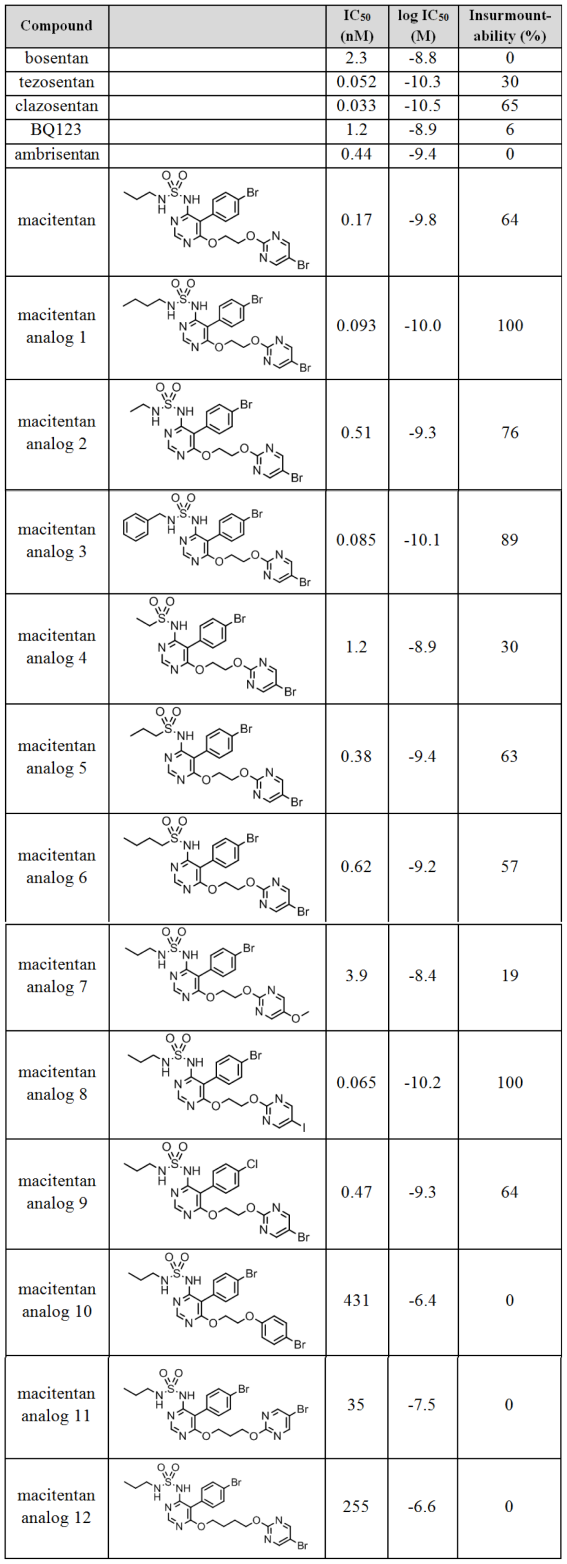
Affinity (IC_50_) and insurmountability of bosentan, tezosentan, clazosentan, ambrisentan, BQ123 and a set of macitentan analogs, measured in ET-1 signaling assays using PASMCs. Insurmountability was determined in IP_1_ accumulation assays using 5 µM ET-1 (20 min stimulation), arithmetic means, n≥3. IC_50_ values were determined in calcium flux assays using 4 nM–12 nM ET-1 (EC_80_), geometric means, n≥4.

Previous experiments in human primary PASMC revealed an almost 20-fold longer ET_A_ receptor occupancy half-life of macitentan compared to ambrisentan and bosentan. This longer ET_A_ receptor occupancy translated into insurmountable antagonism of ET-1-induced ET_A_ receptor signaling by macitentan, but not bosentan or ambrisentan [18]. Here, we extended this analysis and included a set of 12 structural macitentan analogs and two bosentan-like structures, tezosentan [23] and clazosentan [24], to further characterize ET_A_ receptor inhibition kinetics in functional assays (structures in **Fig. 1A** **and** **Figure 2**). To measure target affinity, the inhibitory potency of each compound was assessed using calcium flux assays in human primary PASMC, which predominantly express the ET_A_ receptor subtype. Cells were pre-incubated with compounds for 120 min and the ET-1-induced calcium release (EC_80_: 4–12 nM; determined for each individual experiment) was measured using the fluorescence imaging plate reader (FLIPR). **Fig. 1B** shows a representative ET-1 concentration-response-curve (CRC) in calcium flux assays.

Representative CRCs for macitentan, bosentan and ambrisentan are depicted in **Fig. 1C**. In agreement with previously published data [18], mean IC_50_ values (n = 6) were 0.17 nM (σ_g_ = 1.4) for macitentan, 2.3 nM (σ_g_ = 1.4) for bosentan and 0.44 nM (σ_g_ = 1.4) for ambrisentan. With the exception of macitentan analogs 10 (IC_50_: 431 nM), 11 (IC_50_: 35 nM) and 12 (IC_50_: 255 nM), all investigated compounds were highly potent inhibitors with IC_50_ values ranging from 0.033 to 3.9 nM, as summarized in **Figure 2**.

Insurmountability was assessed using IP_1_ accumulation assays, which were previously used to demonstrate insurmountability of AT_1_ blockers [25]. To this end, PASMC were pre-incubated with ERAs at different concentrations and then stimulated with a very high concentration of ET-1 (5 µM, corresponding to EC_100_ and being ∼1000-fold above the EC_50_). Intracellular IP_1_ levels were determined 20 min after ET-1 addition. **Fig. 1D** shows a representative ET-1 CRC in IP_1_ accumulation assays. **Fig. 1E** shows representative CRC of macitentan, bosentan and ambrisentan against 5 µM ET-1, demonstrating antagonistic efficiency of macitentan and a lack of antagonistic effect of bosentan and ambrisentan up to 1 µM compound concentration. The “percent (%) insurmountability” of the tested ERAs was calculated from the maximal reduction of the ET-1-induced IP_1_ signal achieved by the ERA (maximal ERA concentration 1 µM), compared to the IP_1_ signal obtained without ERA (as shown **Fig. 1E**). The mean (n = 7) % insurmountability obtained for macitentan was 64%+/−15%, whereas bosentan and ambrisentan displayed 0% insurmountability. The mean IC_50_ values (in nM and log M) and the mean insurmountability (in %) of all tested ERAs and the peptidic selective ET_A_ receptor antagonist BQ123 are summarized in **Figure 2**.

When the logIC_50_ values of all tested compounds with IC_50_<5 nM were plotted against the respective % insurmountability (**Fig. 1F**), a linear relationship (R^2^ = 0.88) between the % insurmountability and the logIC_50_ values for macitentan and its structural analogs was revealed. This correlation of antagonistic potency and insurmountability – i.e. of affinity and dissociation rate – suggested that the tested macitentan-like structures share a common ET_A_ receptor interaction mode. In contrast, bosentan, bosentan analogs, ambrisentan and BQ123 showeda different relationship between potency and insurmountability, and compounds with comparable potency to macitentan and its analogs, were considerably less insurmountable indicating rapid ET_A_ dissociation kinetics. As previously published, high affinity compounds with very rapid dissociation kinetics are likely to a display diffusion-controlled binding reaction [20]–[22]. This suggests that the ET_A_ binding of bosentan-like structures and ambrisentan is likely diffusion-controlled and macitentan-like structures which display insurmountability and slower receptor dissociation have a distinct ET_A_ receptor binding mode that is not diffusion-controlled.

### Molecular modeling studies of the interaction between the ET_A_ receptor and its antagonists

**Figure 3:**
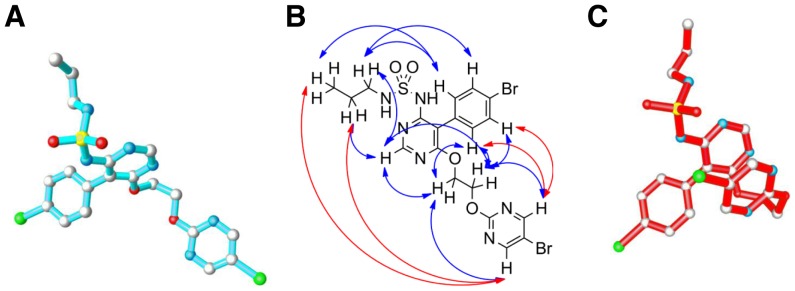
Conformational analysis of macitentan. (A) Structure of macitentan as determined by X-ray structure analysis of crystals obtained from ethyl acetate/hexane shown in “ball and stick” representation. Ball colors are: carbon = grey, nitrogen = blue, oxygen = red, sulfur = yellow, bromine = green. Hydrogens have been omitted for reason of clarity. (B) Structure of macitentan, arrows indicate transfer of magnetization as observed by ^1^H NMR ROESY experiments with macitentan in 0.1 M Na_2_CO_3_ in D_2_O. Blue arrows indicate magnetic interactions observed between constitutionally close neighbors, red arrows indicate magnetic interactions which can only be explained if the molecule assumes a compact conformation. (**C**) Compact conformation of macitentan in aqueous solution as proposed by molecular modeling and corroborated by 2D NMR spectroscopy.

**Figure 4:**
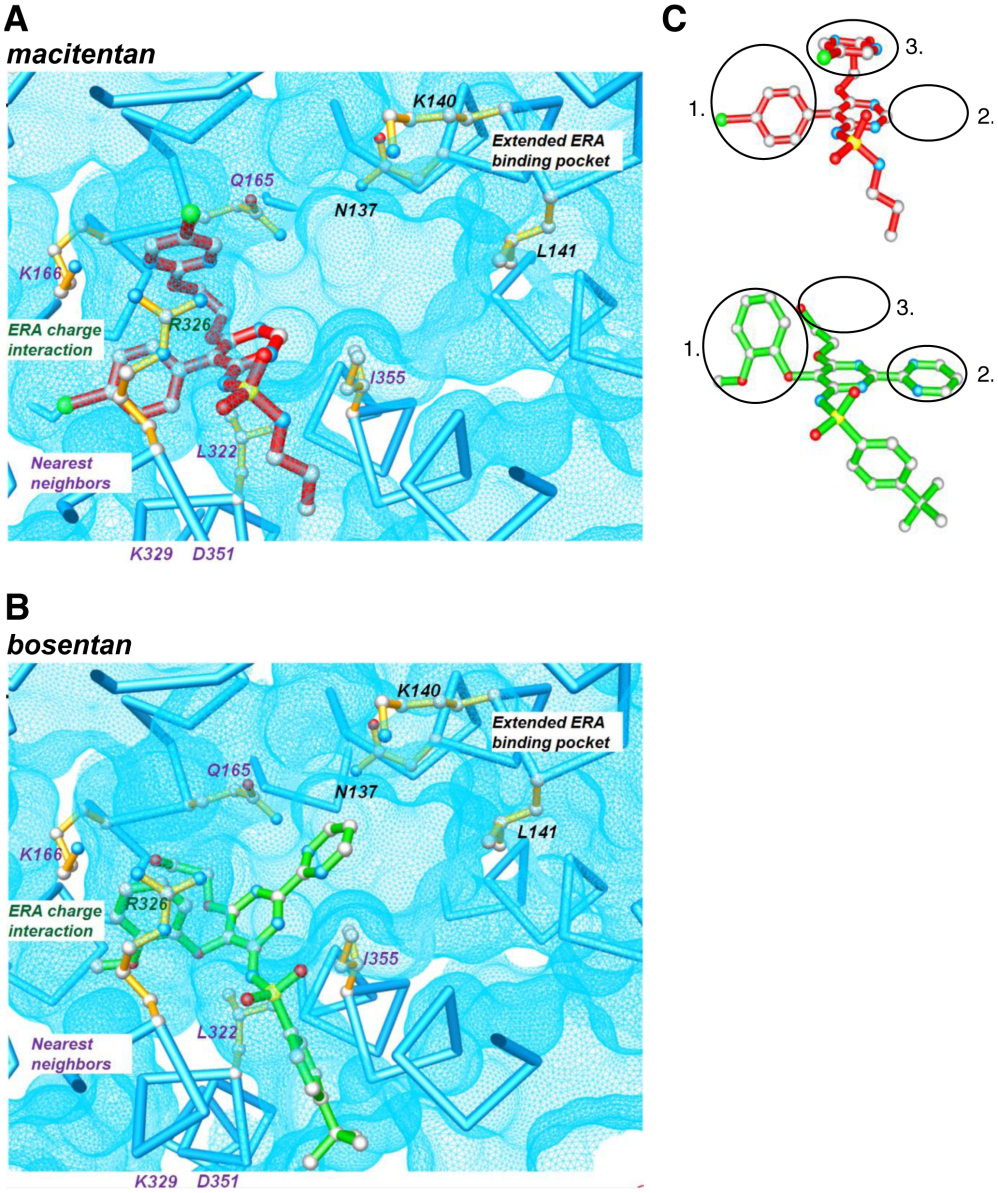
Modeling of macitentan (A) and bosentan (B) binding to the active site of the ET_A_ receptor. The amino acid residues predicted to contact macitentan and/or bosentan are grouped into the categories “nearest neighbors”, “ERA charge interaction” and “extended ERA binding pocket”. (C) Conformations of macitentan (red) and bosentan (green) bound to ET_A_ as proposed by molecular modeling. Relevant structural differences are (1) the different head groups, (2) the length of the central rigid axis spanning over the head group and either one (macitentan) or two (bosentan) pyrimidines, (3) the presence or absence of the 5-bromo-pyrimidine stacking onto the core pyrimidine.

To rationalize this potentially different binding mode a structural level, we built a molecular model of the ET_A_ receptor, based on the recently published orphanin FQ receptor structure [26] (PDB: 4EA3) (see Methods). The ‘open’ conformation of macitentan that was observed in single crystals grown in organic solvents (**Fig. 3A** and [15]) could not be fitted into the expected ligand–binding pocket, which is a sub-pocket of the endothelin binding site in the ET_A_ model (data not shown). However, two dimensional ^1^H NMR spectroscopy (ROESY) of macitentan performed in an aqueous environment demonstrated intramolecular proton interactions (**Fig. 3B**) that suggested a more compact molecular conformation with minimal lipophilic surface exposed to solvent. Our NMR studies also showed that in aqueous solutions containing ≥10% of CD_3_CN this compact conformation is not detectable indicating a relatively high sensitivity of the macitentan conformation towards solvent polarity. These NMR data and calculations performed with the modeling package MOLOC [27] led us to propose a preferred conformation of macitentan in aqueous solution (**Fig. 3C**). **Fig. 4A, B** show macitentan (compact conformation) and bosentan manually docked into the ET_A_ receptor. The neighboring amino acids shaping the binding pocket are Q165, K166, L322, R326, K329, D351 and I355. As an anchor for initial docking, we assumed a charge-charge interaction between the sulfonamide of bosentan and the side chain of R326, as previously published [28]. For macitentan, we assumed an initially similar principle orientation of the sulfamide towards R326.

**Figure 4C** illustrates the structural properties of receptor-bound macitentan and bosentan and highlights the different topologies of these two molecules. The structures of macitentan and bosentan are characterized by a central pyrimidine unit bearing three and four substitutents, respectively. The exchange of the 5-*ortho*-methoxy-phenol unit in bosentan with the 5-*para*-bromo-benzene substituent in macitentan allows a deeper penetration of the latter into the ET_A_ receptor binding pocket (**Fig. 4A, B**). Unlike bosentan, macitentan carries no substituent in the 2-position of the central pyrimidine resulting in a more complete burial of macitentan in the binding pocket. Replacement of the tert.-butyl benzene sulfonamide in bosentan by the propyl sulfamide in macitentan not only decreases the molecular size of this moiety but also increases the pKa of macitentan by about one unit. Finally, the assumed stacking of the 5-bromo-pyrimidine onto the central pyrimidine appears to stabilize the compact conformation of macitentan in aqueous media (**Fig. 3C**) and gives rise to a unique globular overall conformation. This conformation of macitentan allows optimal shape complementarity between inhibitor and binding pocket in the ET_A_ receptor. Due to the reduced acidity of the sulfamide moiety, charge-charge interactions are expected to play a minor role in the receptor binding affinity of macitentan. Hence, the proposed binding forces of macitentan differ from those of bosentan-like molecules (and possibly other ERAs like ambrisentan), which all contain an acidic function and whose binding was proposed to depend on charge-charge interactions [28].

### Functional characterization of point-mutated ET_A_ receptor variants

To verify the structural model and to distinguish the different molecular interaction modes of macitentan, bosentan and ambrisentan, ten point-mutated ET_A_ receptor variants were designed. They incorporated single mutated amino acids which – according to the model – should differentially affect the interaction of the individual ERAs with the ET_A_ receptor (**Fig. 4A, B**).

The R326Q mutant was designed to evaluate a potential charge-charge interaction with the sulfamide moiety in macitentan in comparison with the more acidic sulfonamide of bosentan or the carboxylate of ambrisentan. Six additional amino acids lining the predicted binding pocket, i.e. Q165, K166, L322, K329, D351 and I355, were mutated to probe their role as short-distance ERA interaction partners (‘nearest neighbors’). Finally, three amino acids, N137, K140 and L141 were mutated to probe interactions of amino acids located in the extended vicinity of the presumed ERA binding pocket (‘extended ERA binding pocket’). All mutated amino acids and their suggested role in ERA binding are summarized in **Table 1**.

**Table 1:** Amino acid mutations in ET_A_ receptor predicted to discriminate between ERA binding modes and their functional characterization in ET-1 concentration-response calcium flux assays.

ET_A_ wildtype residue	proposed role in ERA binding	mutation	EC_50_ (nM) of ET-1 [σ_g_]
R 326	ERA charge interaction	R 326 Q	1.8 [1.6]
Q 165	nearest neighbors	Q 165 A	0.73 [1.4]
K 166	nearest neighbors	K 166 A	∼50% signal at 1000 nM
L 322	nearest neighbors	L 322 A	0.63 [1.4]
K 329	nearest neighbors	K 329 M	1.8 [1.4]
D 351	nearest neighbors	D 351 N	0.84 [1.3]
I 355	nearest neighbors	I 355 A	1.8 [1.7]
N 137	extended ERA binding pocket	N 137 A	3.1 [1.1]; Emax∼50%
K 140	extended ERA binding pocket	K 140 I	inactive
L 141	extended ERA binding pocket	L 141 A	inactive
wildtype	wildtype	wildtype	0.68 [1.4]

EC_50_ values were determined in n> = 3 calcium flux assays using isogenic HEK cells expressing the indicated ET_A_ receptor variant. σ_g_: geometric mean.

**Figure 5:**
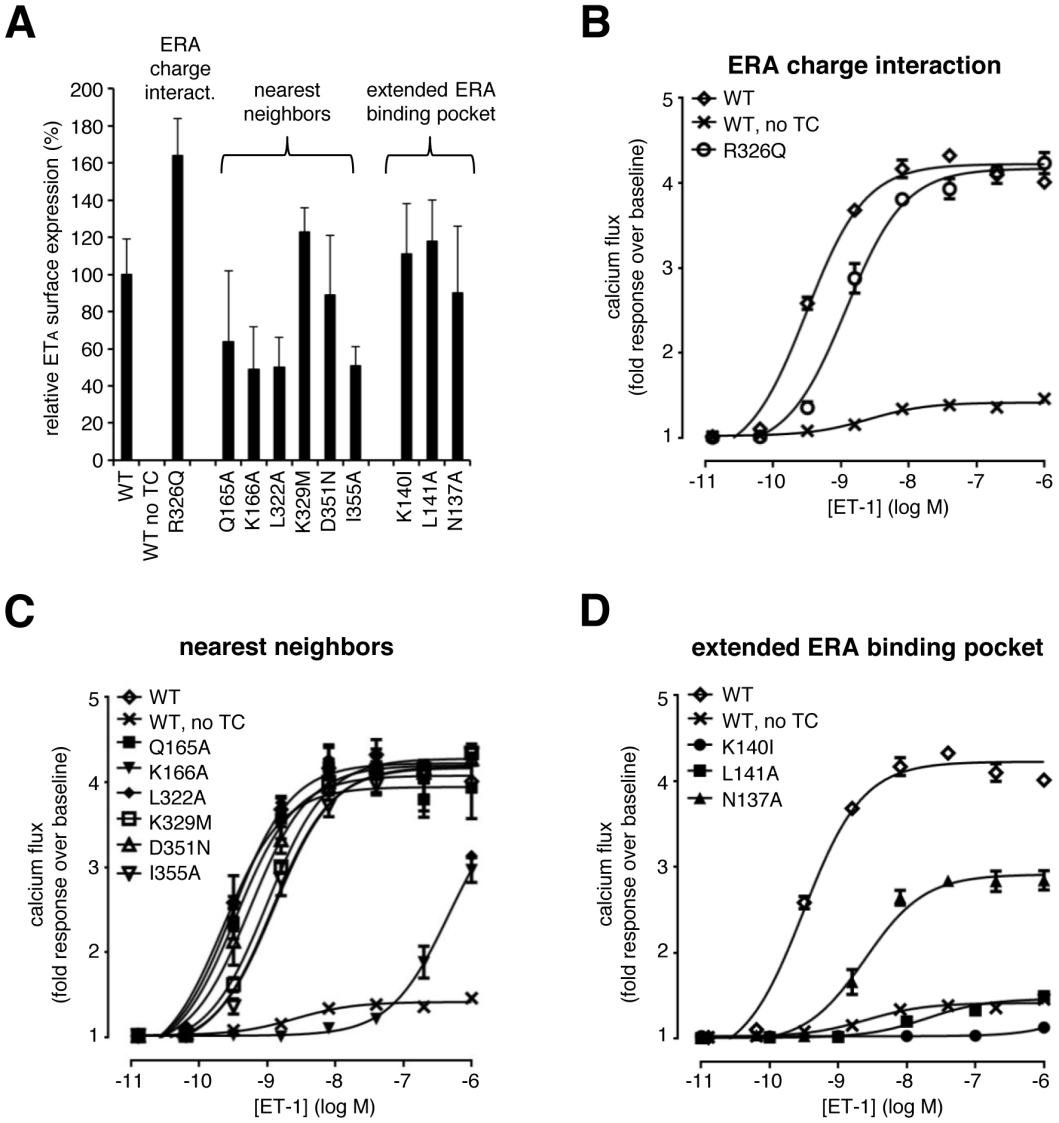
Characterization of isogenic HEK-TRex cell pools expressing point-mutated variants of ET_A_. (A) Cell surface expression levels of ET_A_ receptor variants after induction with tetracycline (100 ng/ml) relative to wildtype ET_A_ receptor (100%) as determined by flow cytometry using anti-FLAG antibodies against the N-terminal FLAG tag. Shown are average expression levels of 4 independent experiments + SEM. (B, C, D) Concentration-response curves for ET-1-induced calcium flux in cell pools expressing the ET_A_ variants. Receptor variants are separated according to the proposed role of the mutated amino acids into the panels “ERA charge interaction”, “nearest neighbors” and “extended ERA binding pocket”. As control, the response of the wildtype receptor with and without tetracycline (TC) induction is shown in every panel. Results of a representative experiment out of n≥3 independent experiments are shown. Values represent averages of duplicates +/− SD.

To analyze ERA interaction with these ten ET_A_ receptor mutants, recombinant isogenic HEK cell pools were generated via homologous recombination. These cells expressed the receptor variants under a tetracycline-inducible promoter. The cell surface expression of the receptor variants after tetracycline induction was determined by flow cytometry using antibodies against the FLAG-tag, which had been added in-frame to the N-terminus. Following tetracycline induction, all receptor variants were present at the cell surface at levels similar to the wild type receptor (**Fig. 5A**). To determine interaction of ERAs with the different receptor variants we employed ET-1-induced calcium flux assays. To this end, we first performed concentration-response experiments with the wildtype receptor (with and without tetracycline induction) and all mutated receptors to evaluate receptor responsiveness to ET-1. Representative CRC are shown in **Fig. 5B, C, D**. Mutant R326Q, which was described to play a key role by forming charge interactions with bosentan [28], did not show any major changes in signal amplitude nor EC_50_ value for ET-1 (mean EC_50_R326Q: 1.8 nM) compared to the wildtype receptor (mean EC_50_wt: 0.68 nM), indicating that this residue had no major role in interactions with the natural ligand (mean EC_50_ values are shown in **Table 1**). **Fig. 5C** shows the ET-1-induced responses of the six ET_A_ receptor variants for which the amino acids that are predicted to line parts of the binding pocket were mutated (‘nearest neighbors’). Clearly, all mutants except variant K166A retained their functionality and showed signal amplitudes and potencies comparable to wildtype receptors with mean potencies of EC_50_Q165A: 0.73 nM, EC_50_L322A: 0.63 nM, EC_50_K329M: 1.8 nM, EC_50_D351N: 0.84 nM and EC_50_I355A: 1.8 nM. Mutant K166A displayed a pronounced shift in ET-1 potency, with significant calcium flux occurring at ≥200 nM ET-1. This indicates an important role for K166 in ET-1 binding or signaling. The ET-1-induced responses of three receptor mutants shaping the vicinity of the macitentan binding pocket is shown in **Fig. 5D** (extended ERA binding pocket). Compared to wildtype receptors, the K140I and L141A variants did not respond to ET-1 at all. The mutant N137A displayed a considerably reduced signal amplitude (∼50% residual signal) and a ∼5-fold lower potency of ET-1 compared to the wildtype receptor, indicating an important role of these three residues for ET-1 binding or signaling.

In summary, the six ET_A_ receptor mutants Q165A, L322A, R326Q, K329M, D351N and I355A did not show major ET-1 response differences versus the wildtype receptor and were therefore employed to analyze ERA binding in functional assays.

### Point mutations R326Q, L322A and I355A differentially affect inhibitory activity of macitentan, bosentan and ambrisentan

Concentration-response experiments were performed in functional assays to quantify ERA potency on cells expressing the six functional ET_A_ mutants or the wildtype receptor. To this end we incubated these cells with macitentan, bosentan or ambrisentan at different concentrations for 120 min followed by addition of 8 nM (EC_90_) of ET-1.

**Figure 6:**
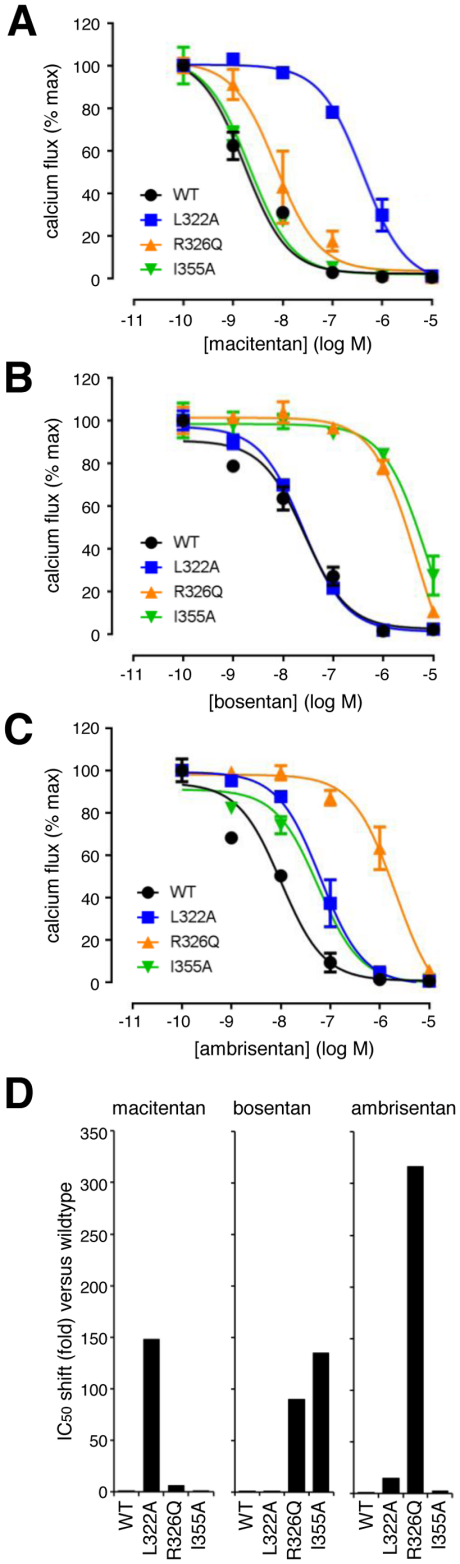
Antagonistic potency of macitentan, bosentan and ambrisentan using three selected point-mutated ET_A_ receptor variants compared to the wildtype receptor. Concentration response curves for macitentan (A), bosentan (B) and ambrisentan (C) in calcium flux assays on the three variants L322A, R326Q, I355A and wildtype using 8 nM ET-1. Results of a representative experiment out of n = 5 independent experiments are shown. Values represent averages of duplicates +/− SD. (D) Graphical representation of the ERA potency loss (IC_50_ shift) on the indicated ET_A_ variant compared to the wildtype receptor. Shifts were calculated from the mean IC_50_ values generated in 5 independent experiments.

Calcium traces were recorded and converted into CRC. **Fig. 6** shows the CRC for macitentan (**A**), bosentan (**B**) and ambrisentan (**C**) generated in cells expressing the wildtype receptor or the L322A, R326Q or I355A receptor variants. The mean IC_50_ values and the IC_50_ shift versus the wildtype receptor are summarized in **Table 2**.

**Table 2:** IC_50_ values of macitentan, bosentan and ambrisentan obtained in calcium flux assays using transgenic cells expressing the indicated point-mutated ET_A_ receptor variants.

ET_A_ variant	Proposed role in ERA binding	IC_50_ (nM) at 8 nM ET-1			IC_50_ shift versus wildtype (fold)		
		macitentan	bosentan	ambrisentan	macitentan	bosentan	ambrisentan
wildtype		1.2	12	2.8	1.0	1.0	1.0
R326Q	ERA charge interaction	7.6	1076	887	6.3	90	317
L322A	nearest neighbors	178	7.3	41	148	0.6	15
K329M	nearest neighbors	0.58	2.3	0.58	0.5	0.2	0.2
D351N	nearest neighbors	1.5	66	4.5	1.2	5.5	1.6
I355A	nearest neighbors	1.1	1608	8.1	0.9	135	2.9
Q165A	nearest neighbors	209	3162	3162	174	264	1129

The right half of the table represents the loss in potency (fold IC_50_ shift) for each compound on each receptor variants compared to the wildtype receptor. IC_50_ values represent geometric means of n = 4–5 independent determinations.

At the wildtype ET_A_ receptor, the three ERAs displayed IC_50_ values of 1.2 nM (macitentan), 12 nM (bosentan) and 2.8 nM (ambrisentan), in good agreement with previous measurements demonstrating approximate equipotency of macitentan and ambrisentan and a 10-fold lower potency of bosentan at the ET_A_ receptor [18]. The potency of macitentan was only slightly affected in mutants R326Q (IC_50_ shift: 6.3) and I355A (IC_50_ shift: 0.9). However, its potency was strongly affected in variant L322A (IC_50_ shift: 148). In sharp contrast, the potency of bosentan was not affected in mutant L322A (IC_50_ shift: 0.6), but was strongly affected in mutants R326Q (IC_50_ shift: 90) and I355A (IC_50_ shift: 135). The potency of ambrisentan was moderately affected in receptor mutant L322A (IC_50_ shift: 15) and I355A (IC_50_ shift: 2.9) and strongly affected in receptor mutant R326Q (IC_50_ shift: 317). In mutant Q165A, antagonistic potency was strongly reduced for all three ERAs (macitentan, IC_50_ shift: 174; bosentan, IC_50_ shift: 264; ambrisentan, IC_50_ shift: 1129). The other two receptor mutants, K329M and D351N, did not significantly affect the potency of any of the ERAs, and IC_50_ shifts were less than 10 in all cases (**Table 2**). **Fig. 6D** graphically represents the compound-specific IC_50_-shift pattern on the mutants L322A, R326Q and I355A for macitentan, ambrisentan and bosentan.

### Confirmation of differential binding mode using structural macitentan and bosentan analogs

**Figure 7:**
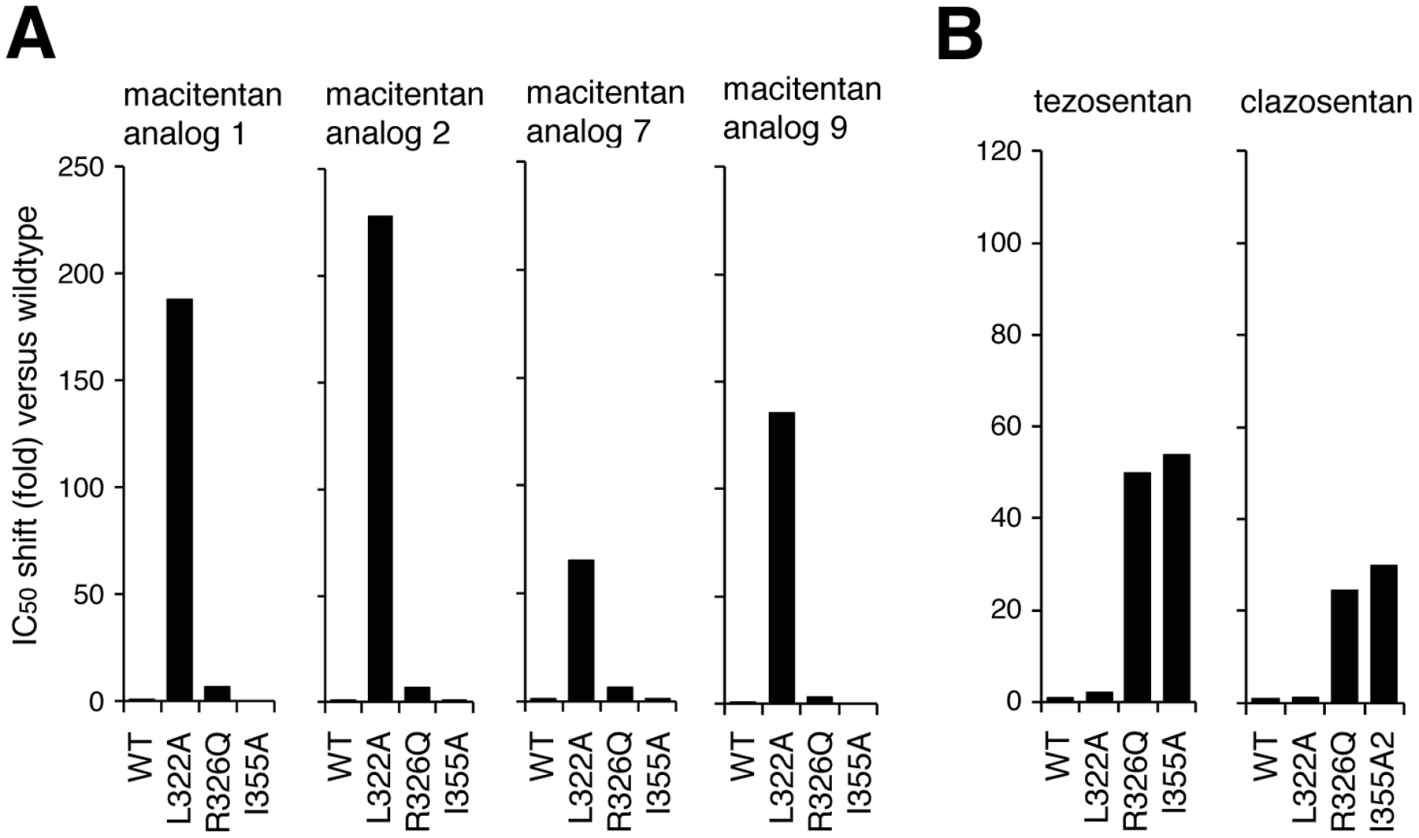
Confirmation of characteristic IC_50_ shifts using macitentan and bosentan analogs. Graphical representation of the potency loss (IC_50_ shift) of macitentan analogs (A) or bosentan analogs (B) on the three ET_A_ mutants L322A, R326Q, I355A compared to the wildtype receptor. Shifts were calculated from the mean IC_50_ values generated in at least 3 independent experiments.

Macitentan, bosentan and ambrisentan displayed different molecular interactions with the ET_A_ receptor as evidenced by different sensitivity to mutations at positions L322, R326 and I355. To further substantiate these differences in amino acid interactions, the potency of macitentan analogs **1, 2, 7** and **9**, and the bosentan analogs, tezosentan and clazosentan, was measured using the described ET_A_ receptor mutants. The antagonistic potencies of these ERAs at the wildtype ET_A_ receptor and the 3 receptor variants and the calculated IC_50_ shifts are shown in **Table 3**. **Fig. 7A and 7B** represent the IC_50_ shift patterns for the tested macitentan and bosentan analogs, respectively. All macitentan analogs showed macitentan-like IC_50_-shift patterns, with strong effects found only in the L322A mutant (IC_50_shifts: 66–228; see **Table 3** and **Fig. 7A**) whereas tezosentan and clazosentan showed a bosentan-like IC_50_-shift pattern, with strong effects observed with mutants R326Q (IC_50_shift: 50/26) and I355A (IC_50_ shift: 54/32), but not in mutant L322A (IC_50_shift: 2.0/1.2). Thus, the differential effects of the mutations L322A, R326Q and I355A were confirmed using structurally related molecules of the corresponding compound series.

**Table 3:** IC_50_ values of four macitentan analogs and two bosentan analogs (tezosentan and clazosentan) obtained in calcium flux assays using transgenic cells expressing the indicated point-mutated ET_A_ receptor variants.

ET_A_ variant	IC_50_ (nM) at 8 nM ET-1				IC_50_ shift versus wildtype (fold)			
	wildtype	L322A	R326Q	I355A	wildtype	L322A	R326Q	I355A
macitentan analog 1	0.17	32	1.2	0.047	1	188	7.1	0.3
macitentan analog 2	4.8	1094	34	3.9	1	228	7.1	0.8
macitentan analog 7	41	2693	268	45	1	66	6.5	1.1
macitentan analog 9	14	1900	46	8.6	1	136	3.3	0.6
tezosentan	0.26	0.53	13	14	1	2.0	50	54
clazosentan	0.085	0.10	2.2	2.7	1	1.2	26	32

The right half of the table represents the loss in potency (fold IC_50_ shift) for each compound on each receptor variant compared to the wildtype receptor. IC_50_ values represent geometric means of n≥3 independent determinations.

## Discussion

### Integration of modeling and mutational data

**Figure 8:**
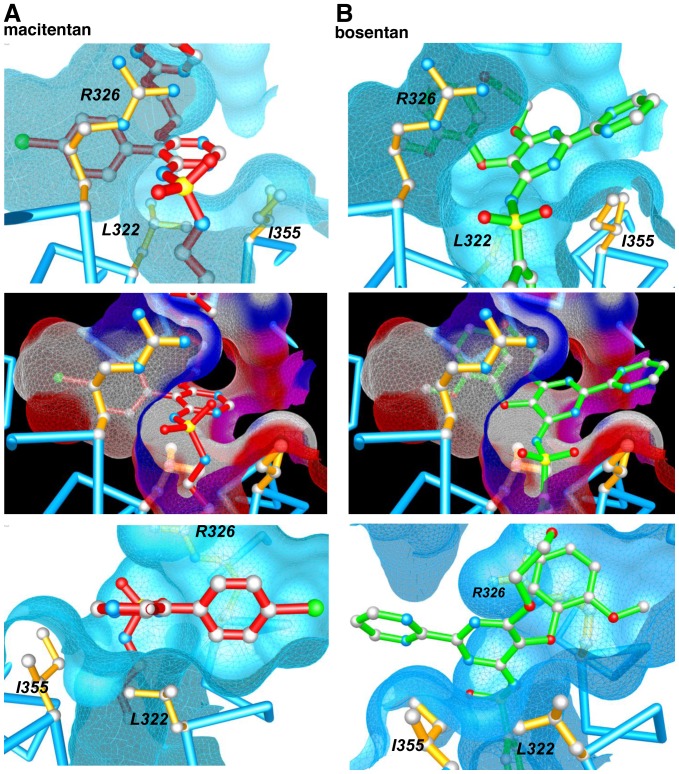
Macitentan (A) and bosentan (B) interacting with the ET_A_ receptor as proposed by mutational experiments and molecular modeling. Three amino acids, L322, R326 and I355, differentially contribute to macitentan and bosentan interaction, causing a hydrophobic and an electrostatic interaction mode, respectively. Two different perspectives of the complexes are shown, one of them including polar surface distribution (red: H-bond acceptor; blue: H-bond donor; pink: H-bond acceptor and donor; white: lipophilic).

The ET_A_ receptor inhibition kinetics of macitentan are characterized by slow dissociation and association rates in comparison to other ERAs of similar affinity [18] which indicated a different binding mode for macitentan compared to other ERAs. Here, we used a combination of molecular modeling and functional studies to characterize the macitentan-ET_A_ receptor interaction site at a molecular level. NMR studies revealed that the lipophilic and only weakly acidic acitentan molecule assumed a compact conformation in aqueous solutions and molecular modeling suggested that this compact conformation allows macitentan to form particularly tight interactions within a spatially confined ET_A_ receptor sub-pocket, potentially not engaging in charge-charge interactions due to its weak acidity. Bosentan, bosentan analogs and ambrisentan, occupy the same pocket, but with a lower fit and - due to their expanded and rigid conformation - molecular interactions extend beyond this sub-pocket into more distal areas. Also, charge-charge interactions are likely contributors to binding. Molecular modeling and physicochemical considerations thus generated a working hypothesis that offered a molecular basis for a different ET_A_ receptor binding mode displayed by macitentan. We next employed functional studies to validate this hypothesis using ten different ET_A_ receptor variants with single amino acid exchanges. Two amino acid changes (K140I and L141A) led to a complete loss of ET-1-induced signaling, whereas the K166A mutation led to a significant reduction of ET-1 potency at the ET_A_ receptor. These results are in line with previous findings showing that the natural ligand ET-1 occupies a large binding pocket within the ET_A_ receptor [28]. All other tested ET_A_ receptor mutants retained their functionality towards ET-1 and were subsequently used to study the binding of ERAs and the ET_A_ receptor. Two of our tested ET_A_ receptor mutants (K329M, D351N) did not affect ERA binding at all, demonstrating that the charges in these two residues are not involved in antagonist binding. In contrast, variant Q165A caused a pronounced loss in potency of macitentan, bosentan and ambrisentan indicating that this residue, located in transmembrane helix 3, is a key contributor to small molecule-receptor binding; however, this amino acid is neither required for ET-1 binding nor signaling. This finding is in good accordance with our molecular model, in which Q165 is in close contact with macitentan and bosentan, thereby restricting spatial motion of both antagonists within the receptor. Interestingly, three receptor mutants, L322A, R326Q and I355A, showed pronounced and differential effects on the potency of macitentan, bosentan and ambrisentan. **Fig. 8A and 8B** show details of this differential interaction of macitentan and bosentan with the three amino acids L322, R326 and I355 in our model of the ET_A_ receptor pocket. R326 was previously identified as an essential component of bosentan - ET_A_ receptor interaction, most likely due to an electrostatic interaction with the acidic sulfonamide group of bosentan (pKa 5.1) [28]. Our studies here confirm and extend this prominent role of R326 to the other charged ERAs, such as ambrisentan (pKa = 3.5), clazosentan (pKa1 = 4.5, pKa2 = 3.3) and tezosentan (pKa1 = 4.4, pKa2 = 4.1). In contrast, the antagonistic potencies of macitentan and its analogs **1, 2, 7** and **9** were not significantly affected in the R326Q variant. These results suggest a significant contribution of the charge-charge interaction involving R326 in the case of bosentan, tezosentan, clazosentan and ambrisentan, and possibly other negatively charged ERAs not tested here. However, for the weakly acidic macitentan (pKa 6.2) charge-charge interaction with R326 does not contribute to the binding affinity. Similarly, the I355A mutant does not affect macitentan but has a major effect on the potency of bosentan, tezosentan and clazosentan. In our model, this amino acid residue forms the outer rim of the hydrophobic binding pocket harboring macitentan (see **Fig. 8A**). The expanded and rigid molecules bosentan (**Fig. 8B**), tezosentan and clazosentan (not shown) cannot be buried entirely in the pocket and protrude into the extended endothelin binding pocket where they interact with I355. Finally, mutant L322A had a significant effect on the potency of macitentan and its analogs and displayed little effect on the potency of bosentan, its structural analogs tezosentan, clazosentan, and on ambrisentan. In our model, L322 forms a significant part of the binding pocket and is involved in tight interactions with macitentan (**Fig. 8A**). In contrast, bosentan-like molecules seem unable to contact L322 as their elongated scaffold forms a contact with I355 (**Fig. 8B**) spanning over L322 at a distance. Thus, macitentan, optimally fills an ET_A_ receptor sub-pocket and this interaction is driven by hydrophobic rather than charge-charge interactions.

Previous work has shown that macitentan, bosentan and ambrisentan are all competitive orthosteric antagonists of ET-1 binding at ET_A_ [18]. While this finding means that ERA binding sites must at least partially overlap with that of ET-1 (excluding allosteric antagonism), it does not allow conclusions on the location of the ERA binding site(s). The present study now adds such information and suggest that the different ERAs tested in this study occupy a similar region defined by the effects of mutations at the closely spaced amino acids R326, L322, I355 and Q165. The ET_A_ binding pocket for the 21-amino acid peptide ET-1 has been proposed to be composed of several sub-sites [29], [30], with the six C-terminal amino acids of ET-1 (His16-Trp21) being accommodated by the most deeply buried sub-site within ET_A_. The amino acids K140, L322, I355 and K166 are proposed as key ET-1 interaction partners within this site [29] which suggests an overlap of this site with the ERA binding region defined in the present study.

### Different binding modes can cause different binding kinetics

Many slowly associating and dissociating drugs operate through a multi-step induced fit mechanism. After rapid formation of an initial loose drug-target complex, subsequent rate-determining slow steps lead to the formation of the final drug-target complex [31]. These subsequent steps (release of water, conformation changes of ligand and/or receptor) can be rather slow and therefore determine association rates. For high-affinity compounds, a slow binding process is accompanied by a slow dissociation (k_off_ = k_on_×K_i_) and leads to increased receptor occupancy times. The macitentan-ET_A_ interaction is characterized by relatively slow association and dissociation kinetics compared to other similarly potent ERAs ([18], and this manuscript). Potential explanations might be the lack of guiding charge-charge interactions and the largely hydrophobic nature of the tight interaction which requires time for water to be released from the binding site. In addition, NMR studies in D_2_O containing increasing amounts of CD_3_CN indicated a relatively high sensitivity of the macitentan conformation towards environment polarity suggesting that macitentan may undergo conformational changes during receptor binding. Thus, the hydrophobic binding mode described in this paper might explain why macitentan and its analogs display a slowed association and dissociation kinetics compared to the other ERAs which are characterized by stronger acidity, lower lipophilicity and charge-dependent binding.

The features described here for macitentan and its interaction with the ET_A_ receptor are not unprecedented for GPCR-antagonist interactions. Recently, an X-ray structure of a GPCR-ligand complex displaying an almost complete burial of the lipophilic ligand vorapaxar in a hydrophobic sub-pocket of the GPCR PAR-1 (protease-activated receptor-1) was described [32]. The authors attributed the experimentally determined sustained binding properties and the insurmountable antagonism of vorapaxar to its precise hydrophobic surface matching of a PAR-1 sub-pocket. The exact opposite was described in an X-ray structure of naltrindole bound to a δ-opioid receptor [33]. The crystal structure of this complex reveals binding of this ligand at the bottom of a wide and solvent-exposed chalice-like pocket, with charge-charge interactions and hydrogen bonds being responsible for high binding affinity. When this opioid ligand is bound, it exposes large surface areas towards the aqueous environment, and existing drug-receptor interactions can be easily weakened by intruding water. For this reason opioid ligands, despite their high affinity, display highly reversible binding, i.e. very short receptor occupancy half-lives, explaining why heroin overdosing is rapidly reversible when treated with the competitive antagonist antidote naloxone [34].

## Conclusions

In summary, macitentan is clearly different from other ERAs, such as bosentan and ambrisentan, in terms of topology and physicochemical properties, and in the present study we show that the unique pharmacological behavior of macitentan - i.e. its increased receptor occupancy time and insurmountability - is paralleled by a class-specific mode of ET_A_ receptor engagement, which is characterized by dominating hydrophobic interactions and minimal electrostatic binding forces.

## Materials and Methods

### Pharmacological compounds

Macitentan and its analogs, ambrisentan, bosentan, tezosentan and clazosentan were synthesized by Actelion Pharmaceuticals (Allschwil, Switzerland). BQ123 was purchased from Sigma (USA).

### NMR Studies

**Figure 9:**
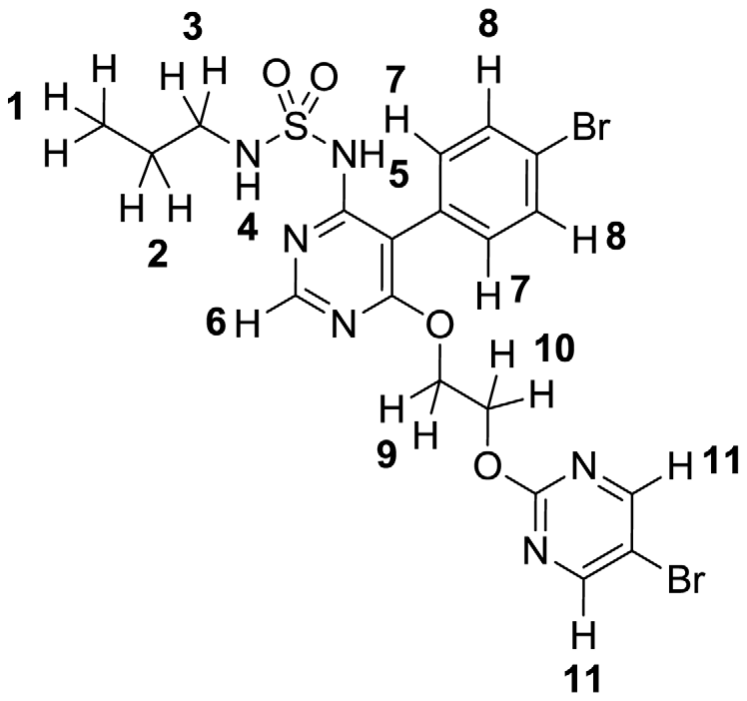
Proton assignment in macitentan as used in NMR interpretation.

Spatial proximity of the various moieties of macitentan's structure (**Figure 9**) was assessed by measuring spatial transfer of magnetization between the corresponding protons by means of two-dimensional Rotating Frame Overhauser Enhancement Spectroscopy (ROESY). Spectra were acquired on a Bruker Avance II, 400 MHz UltraShield, ^1^H (400 MHz), chemical shifts are reported in parts per million (ppm) relative to tetramethylsilane (TMS), and multiplicities are given as s (singlet), d (doublet), t (triplet), q (quartet), quint (quintuplet), h (hextet) or m (multiplet). Approx. 15 mM solutions of macitentan in either 0.1 M Na_2_CO_3_ in D_2_O (pD∼10) or CDCl_3_ were used. Spectral data for macitentan (for proton numbering see below): ^1^H NMR (0.1 M Na_2_CO_3_ in D_2_O): δ 8.16 (s, 2 H, H11), 8.04 (s, 1 H, H6), 7.05–7.12 (m, 2 H, H8), 6.99–7.05 (m, 2 H, H7), 4.24–4.40 (m, 4 H, H9, H10), 2.61 (t, *J* = 6.5 Hz, 2 H, H3), 1.24 (h, *J* = 7.0 Hz, H2), 0.58 (t, *J* = 7.3 Hz, 3 H, H1). ROESY signals: H11→H1, H2, H3, H7, H8, H9, H10; H8→H1, H2, H3, H9, H10, H11; H9, H10→H8, H11; H3→H1, H6, H7, H8, H11; H2→H6, H7, H8, H11; H1→H3, H6, H7, H8, H11. ^1^H NMR (CDCl_3_): δ 8.51 (s, 2 H, H11), 8.48 (s, 1 H, H6), 7.57–7.62 (m, 2 H, H8), 7.16–7.20 (m, 2 H, H7), 6.93 (s, H5), 5.64 (t, *J* = 6.2 Hz, 1 H, H4), 4.72–4.77 (m, 2 H, H9 or H10), 4.62–4.66 (m, 2 H, H9 or H10), 2.98 (q, *J* = 6.7 Hz, 2 H, H3), 1.56–1.66 (m, 2 H, H2), 0.96 (t, *J* = 7.4 Hz, 3 H, H1). ROESY signals: H11→H9, H10; H6→H4, H9, H10; H7→H3, H5, H9, H10; H5→H3, H7; H3→H1.

### Molecular Modeling

An initial C-alpha model of the ET_A_-receptor was built on the structure of the orphanin FQ-receptor (PDB: 4EA3) [26] since this receptor is also agonized by a peptide and - of all to date crystallized GPCRs - showed best sequence identity with ET_A_ (BLAST search [www.pdb.org]: 27% identity, 56% similarity). This identity was considered sufficient to obtain an acceptable model for the ET_A_ receptor [35]. This raw model comprising amino acids T76 to C385 was subsequently optimized using the program MOLOC [27] according to standard procedures [36]. In a next step a full atom model of the ET_A_-receptor was generated. Phi and psi angles were obtained for aligned amino acids from the template receptor. Chi angles were also adopted from the target structure where possible or in case of non-identical amino acids generated by using the most probable value avoiding steric conflicts. In a first optimization step only amino acid side chains were allowed to move while all backbone atoms were kept in fixed positions. This step largely removed repulsive interactions between side chains, further improved chi angles of non-conserved amino acids, and revealed regions with unfavorable interactions. In a following optimization step only C-alpha atoms were kept fixed while all other atoms were allowed to move. In a third round of optimization no atoms were kept stationary but positional constraints were applied to C-alpha atoms. Optimization was continued until MOLOC converged and no further steric conflicts could be detected. Inhibitors were docked manually into the ligand binding site in multiple start positions and conformations followed by a round of optimization with surrounding amino acids allowed to move in each case.

### Generation of recombinant cell pools expressing ET_A_ receptor variants

Isogenic cell pools expressing the different N-terminally FLAG-tagged ET_A_ receptor variants were generated using the Jump-In T-Rex HEK293 cell system (Invitrogen, USA) according to the manufacturer's protocol. This system allows the tetracycline-inducible expression of a gene of interest from a single defined insertion site, so that similar expression levels are obtained from all constructs. In brief, both the retargeting vector containing the gene of interest and the pJTI.R4.Integrase vector were transfected into the Jump-In T-Rex HEK293 parental cell line and selection of recombined isogenic cell pools was performed for 2 weeks in medium containing geneticin (1 mg/ml) and blasticidin (5 µg/ml).

### Cell culture

Jump-In T-Rex HEK-293 cells (HEK-TRex) recombinantly expressing FLAG-tagged ET_A_ receptor variants were cultivated in DMEM, GlutaMAX-I, containing 4.5 g/l D-glucose, pyruvate, 1% non-essential amino acids, 10% dialyzed FCS, 1000 µg/ml geneticin, 100 U/ml penicillin, and 5 µg/ml blasticidin. PASMC were purchased from Lonza (CC-2581) and cultivated in Lonza Clonetics SmBM growth medium (CC-3181) with SmGM-2 SingleQuot supplements and 5% FCS (CC-4149). PASMC of passage 3 to 5 were used for all experiments.

### Intracellular calcium flux measurements using human PASMC

PASMC were seeded in growth medium at 8,000 cells/well into 384-well black clear-bottom plates (Greiner, Germany) and incubated overnight at 37°C in 5% CO_2_. Then, cell culture medium was exchanged by 50 µL/well of dye buffer [Hank's Balanced Salt solution (HBSS), 0.1% bovine serum albumin (BSA), 20 mM HEPES, 0.375 g/L NaHCO_3_, 5 mM probenecid and 3 µM of fluo-4 AM (Life Technologies)]. The cell plates were incubated for 1 h at 37°C in 5% CO_2_, and then equilibrated at room temperature for at least 30 min. For the determination of the inhibitory potency (IC_50_) of the endothelin receptor antagonists, within the Fluorescent Imaging Plate Reader (FLIPR Tetra, Molecular Devices), cells were supplemented with 10 µL of 6× concentrated antagonist dilution series prepared in assay buffer (HBSS containing 0.1% BSA, 20 mM HEPES, 0.375 g/L NaHCO_3_, pH 7.4) covering the final concentration range from 0 nM to 100 nM or 0 nM to 1000 nM. The final assay concentration of DMSO was 0.1%. After 120 min at room temperature, cells were stimulated with ET-1 by the addition of 10 µL of 7× concentrated ET-1 in assay buffer to obtain a final concentration of EC_80_ (4 nM–12 nM, determined on the day of every experiment). Calcium flux was monitored for 3 min. FLIPR traces were normalized by trace alignment at the last time point before agonist addition (ScreenWorks software, Molecular Devices, USA). Then, relative fluorescence units (RFU) of the maximum signal per well were exported and used by the proprietary IC_50_ Witch software to calculate IC_50_ values [settings: fixed minimum (1 µM macitentan) and curve-intrinsic maximum].

### IP_1_ measurements

All measurements were performed using the IP-One HTRF kit (Cisbio, France) following the manufacturer's protocol. PASMC were seeded at 10,000 cells/20 µµL/well into 384-well, white, small-volume tissue culture plates (Greiner, Germany,) and incubated overnight at 37°C in 5% CO_2_. Then, medium was removed and 5 µL/well of assay buffer (HBSS containing 0.1% BSA, 20 mM HEPES, 0.375 g/L NaHCO_3_, 50 mM LiCl, pH 7.4) were added. Then, cells were supplemented with 5 µL/well of a 2× concentrated dilution series (3-fold) of antagonists in assay buffer, covering a final concentration range of 0 nM to 370 nM, and incubated for 120 min at 37°C in 5% CO_2_. Then, 2.5 µL/well of 5× concentrated solutions of ET-1 in assay buffer giving a final concentration of 5 µM were added followed by an incubation at 37°C/5% CO_2_ for 20 min. Cells were lysed by the addition of 2.5 µL/well of conjugate-lysis buffer. Then, 2 µL/well of IP_1_-d2 conjugate and 3 µL/well of anti-IP_1_-cryptate terbium were added. After 60 min incubation at room temperature, the assay plates were excited at 337 nm and the ratio of emitted light at 665 nm and 620 nm was recorded using a PHERAstar microplate reader (BMG Labtech, Germany). 665 nm/620 nm emission ratios were translated into IP_1_ concentrations via an on-plate calibration curve generated with known concentrations of IP_1_. IP_1_ concentration values were exported into GraphPadPrism software and the antagonistic concentration-response curves (CRC) were fitted by three-parameter non-linear regression, giving compound specific values for the upper plateau (no inhibition) and lower plateau (maximal inhibition). The degree of insurmountability (%) was calculated as (upper plateau−lower plateau)/upper plateau×100%. To compare results from independent experiments, insurmountability values were normalized against the fully insurmountable compound macitentan analogs 1 or 8 (100% insurmountable). Compounds having an insurmountability of less than 15% in an individual experiment were classified as surmountable (0% insurmountable).

### Intracellular calcium flux measurements using recombinant HEK-TRex cells expressing different ET_A_ receptor variants

Recombinant HEK cells expressing the human ET_A_ variants were seeded in growth medium (in presence or absence of 100 ng/mL tetracycline) at 12,000 cells/well into poly-l-lysine 384-well black clear-bottom plates (Greiner, Germany) and incubated overnight at 37°C in 5% CO_2_. Then, the growth medium was exchanged with 50 µl/well of dye buffer (HBSS with 20 mM HEPES, 0.375 g/l NaHCO_3_, and 3 µM fluo-4 AM). Cells were incubated for 1 h at 37°C in 5% CO_2_, then medium was exchanged with 50 µl/well assay buffer (HBSS with 0.1% BSA, 20 mM Hepes, 0.375 g/l NaHCO_3_) followed by equilibration at room temperature for at least 30 min. For the determination of the inhibitory potency (IC_50_) of the endothelin receptor antagonists, within the Fluorescent Imaging Plate Reader (FLIPR Tetra, Molecular Devices, USA), cells were supplemented with 10 µl of 6× concentrated antagonist dilution series prepared in assay buffer covering the final concentration range from 0 nM to 10 µM (0.1% DMSO final assay concentration). After a 120-min incubation at room temperature, cells were stimulated by the addition of 10 µl of 7× concentrated ET-1 in assay buffer to obtain the final assay concentration of 8 nM (EC_90_). Calcium flux was monitored for 3 min. For the IC_50_ calculations, FLIPR traces were normalized by trace alignment at the last time point before agonist addition (ScreenWorks software, Molecular Devices, USA). Then, relative fluorescence units (RFU) of the maximum signal per well were exported to the proprietary IC_50_ Witch software (Actelion, Switzerland) and used to calculate IC_50_ values [settings: fixed minimum (0 nM ET-1) and curve-intrinsic maximum]. For the determination of ET-1 potency and efficacy at the different receptor variants, cells stained with fluo-4 were stimulated with 10 µl of a 7× concentrated dilutions series of ET-1 in assay buffer and calcium responses were recorded and processed as described above. To optimally compare response amplitude and ET-1 potency for the different cell pools, CRCs were displayed as response over baseline. Potencies were calculated using the IC_50_ Witch software (settings: intrinsic minimum and intrinsic maximum).

### Flow cytometry

Recombinant HEK cells expressing the FLAG-tagged human ET_A_ variants were seeded at 10^6^ cells per well (in presence or absence of 100 ng/ml tetracycline) into 6-well plates in growth medium and incubated overnight at 37°C in 5% CO_2_. Then, the cells were washed with PBS and detached with cell dissociation buffer (Life Technologies, USA). FLAG-hET_A_ receptors were stained with mouse anti-FLAG M2 antibodies (Sigma, USA), diluted in PBS/2 mM EDTA/0.5% fatty acid free BSA (Calbiochem, Germany). As secondary antibody Alexa Fluor 488 goat anti-mouse IgG (Life Technologies, USA) was used. Then, mean fluorescence intensities (MFI) of the propidium iodide-negative cells were recorded using a FACSAria IIu flow cytometer (BD Biosciences, USA). Surface receptor expression was calculated by subtracting the MFI of the non-induced cell pools from the MFI of the tetracycline-induced cells pools and then comparing this value to the value obtained for the wildtype-expressing cells (100%).

### Statistics

Arithmetic (SD) or geometric (σ_g_) standard deviations were calculated according to standard procedures and are indicated in the table and figure legends.
